# Astaxanthin protects astrocytes against trauma-induced apoptosis through inhibition of NKCC1 expression via the NF-κB signaling pathway

**DOI:** 10.1186/s12868-017-0358-z

**Published:** 2017-05-10

**Authors:** Mingkun Zhang, Zhenwen Cui, Hua Cui, Yong Wang, Chunlong Zhong

**Affiliations:** 1 Department of Neurosurgery, Ren Ji Hospital, School of Medicine, Shanghai Jiao Tong University, Shanghai, 200127 China; 2 Department of Neurosurgery, East Hospital, Tongji University School of Medicine, 150 Jimo Road, Shanghai, 200120 China; 3 Department of Neurosurgery, The Affiliated Hospital of Qingdao University, Qingdao, 266005 China

**Keywords:** Astaxanthin, Traumatic brain injury, Apoptosis, Astrocyte, Na^+^–K^+^–2Cl^−^ co-transporter-1, NF-кB

## Abstract

**Background:**

Astaxanthin (ATX) is a carotenoid pigment with pleiotropic pharmacological properties that is seen as a possible drug for treating cerebral ischemic injury and subarachnoid hemorrhage. Na^+^–K^+^–2Cl^−^ co-transporter-1 (NKCC1), an intrinsic membrane protein expressed by many cell types, is activated by various insults, leading to the formation of cell swelling and brain edema. We previously established that ATX attenuated brain edema and improved neurological outcomes by modulating NKCC1 expression after traumatic brain injury in mice. This paper explored the molecular mechanism of ATX-mediated inhibition of NKCC1 utilizing an in vitro astrocyte stretch injury model.

**Results:**

Stretch injury in cultured astrocytes lowered cell viability time-dependently, which was substantially reducing by pretreating with ATX (50 μmol/L). Stretch injury increased Bax level and cleaved caspase-3 activity, and decreased Bcl-2 level and pro-caspase 3 activity, resulting in the apoptosis of astrocytes. Additionally, stretch injury substantially raised the gene and protein expressions of interleukin (IL)-1β, IL-6, and tumor necrosis factor (TNF)-α and prompted the expression and nuclear translocation of NF-κB. Pretreatment with ATX remarkably prevented the trauma-induced initiation of NF-κB, expressions of pro-inflammatory cytokines, and cell apoptosis. Moreover, stretch injury markedly elevated the gene and protein expression of NKCC1, which was partly blocked by co-treatment with ATX (50 µmol/L) or an NF-κB inhibitor (PDTC, 10 µmol/L). Cleaved caspase-3 activity was partially reduced by PDTC (10 µmol/L) or an NKCC1 inhibitor (bumetanide, 50 µmol/L).

**Conclusions:**

ATX attenuates apoptosis after stretch injury in cultured astrocytes by inhibiting NKCC1 expression, and it acts by reducing the expression of NF-κB-mediated pro-inflammatory factors.

## Background

Cerebral edema is secondary to primary lesions or dysfunction of the nervous system after traumatic brain injury (TBI). One outcome of edema is the development of increased intracranial pressure, which can result in brainstem compression, brain herniation, coma, and failure of the respiratory and/or cardiovascular systems [1]. Edema, as well as the complications associated with it, is responsible for up to 50% of the mortality in all victims of TBI [2]. Despite the fact that vasogenic as well as cytotoxic mechanisms participate in TBI-related brain edema, cytotoxic edema (intracellular swelling) prevails in the early phase (2–24 h) of TBI, at which point astrocytes are the major cell type affected [3]. Although it has been documented over the course of several years as a critical post-TBI issue, cerebral edema treatment strategies are currently limited, and a majority of them are not effective.

Astaxanthin (ATX), a lipophilic compound extracted from crustaceans, algae, shellfish, and a variety of plants [4], has strong biological effects, including antioxidative, anti-inflammatory, antitumor, and immunomodulatory activities [5, 6]. Recently, studies have shown that the antioxidant and anti-inflammatory effects of ATX are beneficial for the treatment of central nervous system diseases without causing any side effects or toxicity [7–10]. Treatment with ATX after subarachnoid hemorrhage significantly downregulated increased nuclear factor kappa B (NF-кB) activity and the expression of inflammatory cytokines via messenger RNA transcription and protein synthesis, leading to the amelioration of blood-brain barrier disruption, cerebral edema, neuronal degeneration, and neurological dysfunction [8]. We previously reported that ATX attenuated brain edema and improved neurological outcomes in TBI mice [11]. Yet, the molecular mechanisms underlying the ATX-dependent inhibition of the cerebral edema in TBI remain poorly understood.

The Na^+^–K^+^–2Cl^−^ co-transporter-1 (NKCC1) is an intrinsic membrane protein expressed in a plethora of cell types, including astrocytes, cortical and cerebellar neurons, oligodendrocytes, brain capillary endothelial cells, and epithelial cells of the choroid plexus [12, 13]. It plays a vital part in cell volume homeostasis by transporting chloride, sodium and/or potassium ions, across the plasma membranes of cells. The inappropriate activation of NKCC1 is implicated in the formation of cell swelling and brain edema caused by various insults, including TBI [12, 14–16], ischemic stroke [17–19], hemorrhagic stroke [13], and tumors [20, 21]. Edema was markedly reduced by the cotransporter inhibitor bumetanide or genetic deficiency [13]. The goal of this study was to elucidate whether the neuroprotective effects of ATX are associated with alterations of NKCC1 levels using an in vitro astrocyte stretch injury model. Furthermore, we determined whether ATX administration decreased NKCC1 expression to clarify the possible regulatory pathway.

## Methods

### Materials

ATX (Santa Cruz Biotechnology, Santa Cruz, CA, USA, 98% pure) was dissolved in dimethyl sulfoxide (DMSO); the DMSO content in every group was 0.1%. Pyrrolidine dithiocarbamate (PDTC), a NF-кB inhibitor, was purchased from Beyotime (Jiangsu, China) and used at a concentration of 10 μmol/L. Bumetanide, a NKCC1-specific inhibitor, was purchased from Sigma-Aldrich (St. Louis, MO, USA) and used at a concentration of 50 μmol/L [22]. Anti-NKCC1, anti-glial fibrillary acidic protein (GFAP) and anti-NF-кB/p65 antibodies were purchased from Millipore (Billerica, MA, USA) and Santa Cruz Biotechnology, respectively. Anti-pro caspase 3 antibody was purchased from Abcam (Cambridge, MA, USA). Antibodies against Bcl-2, Bax, cleaved caspase-3, IL-1β, IL-6, TNF-α, β-actin, β-tubulin, and Histone H3 were purchased from Cell Signaling Technology (Danvers, MA, USA).

### Astroglial cell culture

All experimental protocols were performed in accordance with the guidelines of the Institutional Ethics Committee of Renji Hospital, Shanghai Jiao Tong University School of Medicine. Primary cultures of astrocytes were isolated from the cerebral cortices of neonatal C57BL/6 mouse pups (P0-P3). After the meninges and blood vessels were carefully removed under a dissecting microscope, cerebral tissues were minced mechanically and incubated in 0.25% trypsin for 10 min. Isolated cells were grown until confluent in astrocyte medium [Dulbecco’s modified Eagle’s medium supplemented with 4.5 g/L d-glucose, 4 mmol/L l-glutamine, 3.7 g/L sodium bicarbonate, with 10% fetal bovine serum, penicillin (100 units/ml) and streptomycin (100 mg/ml)]. Greater than 90% of cells in these cultures were GFAP-positive. The cells were maintained in a humidified atmosphere at 37 °C with 95% air–5% CO_2_. The culture medium was renewed after 24 h, and then every 2 days thereafter.

### Cell injury

After 7-to-10 days, confluent cultures of astrocytes grown in BioFlex^®^ Plates were injured using a model 94A Cell Injury Controller [23]. Briefly, a 50-ms pulse of compressed air was used to rapidly deform the Silastic membrane of adherent astrocytes to produce a 7.5-mm membrane deflection, which corresponds to a 54% membrane stretch and severe cell injury. After the insult, cells were placed in a 95–5% air–CO_2_ incubator at 37 °C for various times. The cells in the ATX-treated group were incubated with ATX for 2 h before injury and for an additional 24 h without altering the culture medium. Cells treated with PDTC or bumetanide were performed identically. The control-cultured cells were incubated over 24 h with culture medium. It is important to note that no hypoxic or ischemic conditions were imposed on the injured cells in these experiments.

### Cell viability assay

A Cell Counting Kit-8 (CCK-8, Beyotime Biotechnology, Jiangsu, China) was used to quantitatively evaluate cell viability according to the manufacturer’s instructions. In short, 90 ml of cell suspension was incubated along with 10 ml of 2-(2-methoxy-4-nitrophenyl)-3-(4-nitrophenyl)-5-(2,4-disulfophenyl)-2*H*-tetrazolium and monosodium salt (WST-8) solution for 4 h at 37 °C in a 5% CO_2_ atmosphere before terminating the assay. The optical density (OD) values were measured at 450 nm using a microplate reader (680; Bio-Rad, Hercules, CA, USA).

### In situ cell death detection

Apoptotic cells were investigated by TdT-dUTP nick-end labeling (TUNEL) assays with a one-step in situ cell death detection kit (Roche, Germany) based on the manufacturer’s directions. After injury, astrocytes were fixed with freshly prepared 4% paraformaldehyde solution in phosphate buffered saline (PBS) for 60 min at 4 °C, washed with fresh PBS twice for 5 min, and then incubated with 0.1% Triton X-100 for 2 min on ice. The cells were then incubated with 50 μl of TUNEL reaction mixture in a humidified atmosphere for 1 h at 37 °C in the dark, and then washed 3 times with PBS. Cell nuclei were stained with 4′,6-diamidino-2-phenylindole (DAPI) (1:5000, Carlsbad, CA, USA) for 5 min, and washed in PBS for 5 min at room temperature. Cell images were captured by immunofluorescent microscopy. The cells that appeared with red fluorescence were deemed to be apoptotic.

### Total RNA extraction and relative quantitative real time-PCR analysis

Total RNA was removed from cell cultures with TRIzol reagent (Invitrogen) based on the company’s instructions. Extracts were treated with Rnase-free DNase to eliminate any residual genomic DNA. Reverse transcription was performed using a One Step SYBR^®^ Prime-Script™ PLUS RT-PCR Kit (Takara Bio Inc., Shanghai, China). The oligonucleotide primers used to amplify the target genes were as follows: GAPDH, 5′-AGCCACATCGCTCAGACAC-3′ (forward) and 5′-GCCCAATACGACCAAATCC-3′ (reverse); interleukin (IL)-1β, 5′-ATGGGATAACGAGGCTTATGTG-3′ (forward) and 5′-CAAGGCCACAGGTATTTTGTC-3′ (reverse); IL-6, 5′-ACTTGCCTGGTGAAAATCAT-3′ (forward) and 5′-CAGGAACTGGATCAGGACTT-3′ (reverse); tumor necrosis factor (TNF)-α, 5′-TCAGCAAGGACAGCAGAGG-3′ (forward) and 5′-CAGTATGTGAGAGGAAGAGAACC-3′ (reverse); NKCC1: 5′-TGATTCCACTTCCTTTATTGCAG-3′ (forward) and 5′-TTAATGAG TTGAGCTCCGGTGA-3′ (reverse); NF-κB, 5′-TATTTCAACCACAGATGGCACT-3′ (forward) and 5′-AGCAAAGGCAATACATACACTT-3′ (reverse). All assays were performed 3 times; the results were normalized by GAPDH as an internal control to calculate the ΔCt. PCR amplification was done with the parameters: 95 °C for 40 s, 55 °C for 45 s, and 72 °C for 50 s. The relative gene expressions were determined with SDS software (Applied Biosystems, Carlsbad, CA, USA) following 40 cycles.

### Extraction of nuclear and cytosolic fractions

For protein extraction and isolation, a nuclear and cytoplasmic protein extraction kit (Beyotime, Jiangsu, China) was used to separate nuclear and cytoplasmic proteins according to the manufacturer’s recommendations. In short, the cells were washed 3 times with PBS after treatment, then scraped, and finally gathered by centrifugation at 1500 × *g* for 5 min. Cell pellets were lysed on ice for 15 min with 200 ml extraction buffer A. After, extraction buffer B was included and samples were vortexed for 30 s at 4 °C. After the centrifugation of samples at 12,000×*g* for 5 min, supernatants were collected and used as cytoplasmic fractions. Pellets were then lysed for 30 min on ice in 50 ml of nuclear extraction buffer with brief vortexing. After another centrifugation step at 12,000×*g* for 5 min, supernatants were collected and used as nuclear fractions.

### Western blot analysis

Proteins were extracted from cell cultures after treatment, and protein concentrations were quantified using a bicinchoninic acid protein assay kit (Pierce Biotechnology, Rockford, IL, USA). Then, 30 μg of protein was loaded onto a 6 or 8% polyacrylamide gel for electrophoresis and then electrotransferred onto a polyvinylidene difluoride membrane (Millipore, Bedford, MA, USA), which was then blocked with 5% non-fat milk followed by incubation with the appropriate primary antibodies at 4 °C overnight. Following washing the blots 3 times, the membranes were incubated with HRP-anti-rabbit IgG (HangZhouHuaAn Biotechnology, Zhejiang, China) for 1 h at room temperature. Immunoreactive proteins were identified via an ECL chemiluminescence system (Pierce Biotechnology). The protein levels were determined by densitometry using Image J1.43 software (National Institutes of Health, Bethesda, MD, USA).

### Immunofluorescence

Astrocytes were seeded in BioFlex^®^ Plates and pretreated with 50 μmol/L ATX or 10 μmol/L PDTC for 2 h before the induction of injury. Following 24 h of incubation, the cells were fixed in 4% paraformaldehyde for 15 min, permeabilized with 0.3% Triton X-100 solution for 20 min, and then obstructed in 10% bovine serum albumin for 30 min. Then, the cells were treated overnight at 4 °C with primary rabbit anti-NKCC1 (1:50) antibody and mouse anti-glial fibrillary acidic protein (1:200). After being washed, the sections were incubated with secondary antibody, Alexa Fluor 488 donkey anti-rabbit IgG (1:500, Invitrogen) and Alexa Fluor 594 donkey anti-mouse IgG (1:500, Invitrogen), for 1 h. DAPI (1;5000, Invitrogen) was employed to counterstain the cell nuclei. A Leica confocal laser-scanning microscope (Leica, Wetzlar, Hesse, Germany) was used to acquire confocal microscopic images.

### Statistical analysis

Statistical analyses were performed using GraphPad Prism (version 5.01, GraphPad Software Inc., San Diego, CA, USA). Data are presented as the mean ± standard error of the mean (SEM) and differences among groups were assessed by one-way analysis of variance followed by the Student–Newman–Keuls test. Statistical significance was set when p < 0.05.

## Results

### Effects of ATX on astrocyte viability induced by injury

**Fig. 1:**
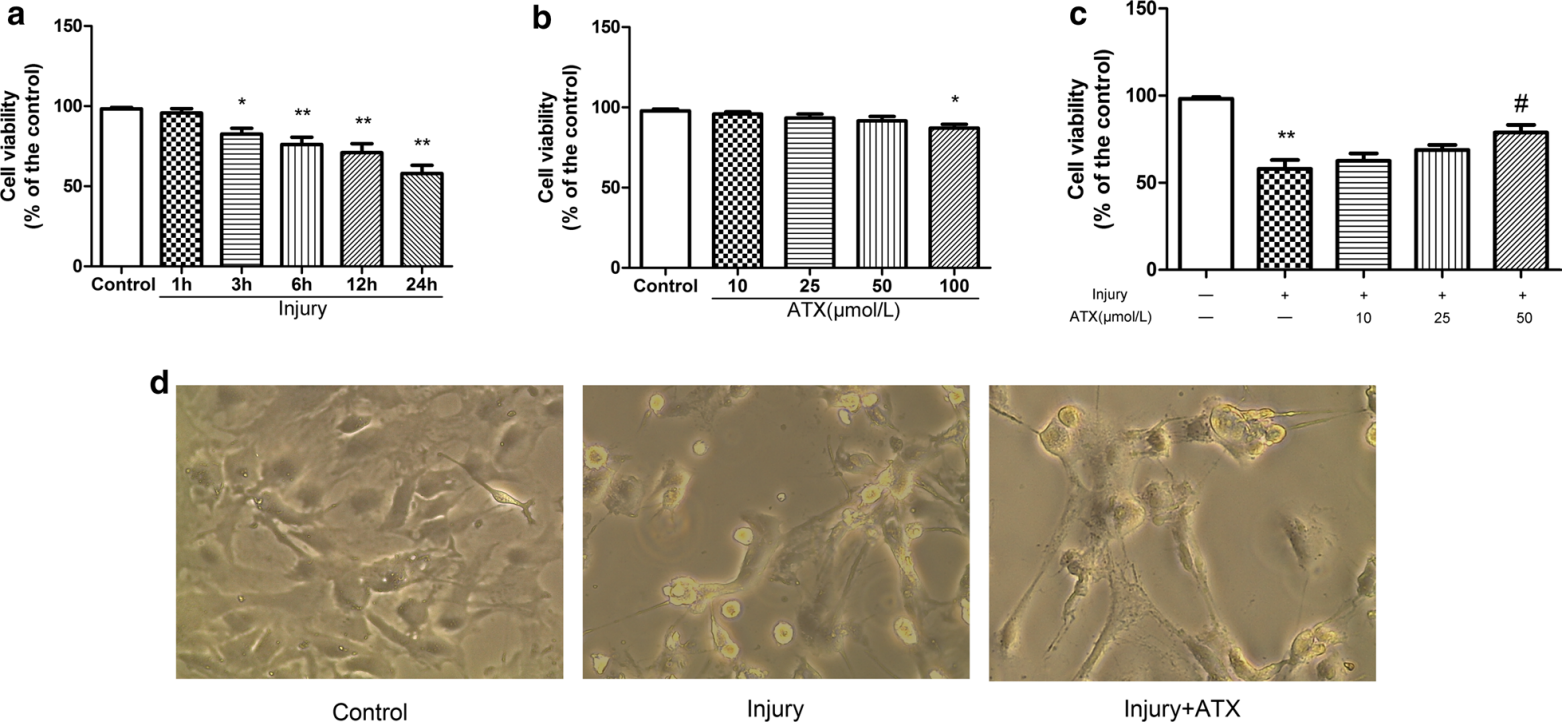
The protective effects of ATX on astrocyte viability after stretch injury. **a** Astrocytes underwent stretch-induced cell injury. Cell viability at 1, 3, 6, 12, and 24 h after injury was tested by CCK-8 assays. **b** Astrocytes were treated with different concentrations of ATX (10, 25, 50 or 100 μmol/L) for 24 h. Cell viability was estimated using CCK-8 assays. **c** Astrocytes were pretreated with 10, 25, or 50 μmol/L ATX for 2 h and then injured and incubated for 24 h. CCK-8 assays were performed to evaluate cell viability. **d** Astrocytes were incubated with 50 μmol/L ATX for 2 h prior to injury for another 24 h, and then morphological changes were analyzed (×200). *ATX* astaxanthin. Mean ± SEM (n = 3). *p < 0.05; **p < 0.01 versus the control group; ^#^ *p* < 0.05 versus the injury group

To determine the influence of injury and positive effect of ATX on injured astrocytes, cells with stretch-induced cell injury were treated with 10, 25, 50, or 100 μmol/L of ATX for 24 h. CCK-8 assays were conducted to investigate cell viability. The viability of astrocytes at 1, 3, 6, 12 and 24 h after injury was 95.68 ± 2.72, 83.93 ± 3.68% (*p* < 0.05), 76.11 ± 4.43% (*p* < 0.01), 70.97 ± 5.54% (*p* < 0.01), and 57.90 ± 5.10% (*p* < 0.01) of the control values, respectively (Fig. 1a). This suggested that cell viability slowly diminished following injury and 24 h was selected for the experiments. The viability of cells incubated with ATX at 10, 25, 50 and 100 μmol/L for 24 h was 96.52 ± 1.69, 93.28 ± 2.53, 91.55 ± 2.77, and 87.04 ± 2.40% (*p* < 0.05) compared to the untreated control, respectively (Fig. 1b). The outcomes revealed up to 50 μmol/L of ATX did not have an impact on the viability of the astrocytes and this non-cytotoxic ATX concentration was implemented in every one of the experiments in our study. The decrease in the viability of astrocytes after injury was significantly alleviated by pretreatment with 50 μmol/L of ATX compared with controls (Fig. 1c, 57.90 ± 5.10% vs 78.75 ± 4.43%, *p* < 0.05). As viewed in Fig. 1d, stretch-induced cell injury triggered vivid morphological changes characteristic of astrocyte damage, while pretreatment with ATX for 2 h before injury somewhat reduced injury-induced cytotoxicity and cell damage.

### ATX inhibits the apoptotic cell death of astrocytes after injury

**Fig. 2:**
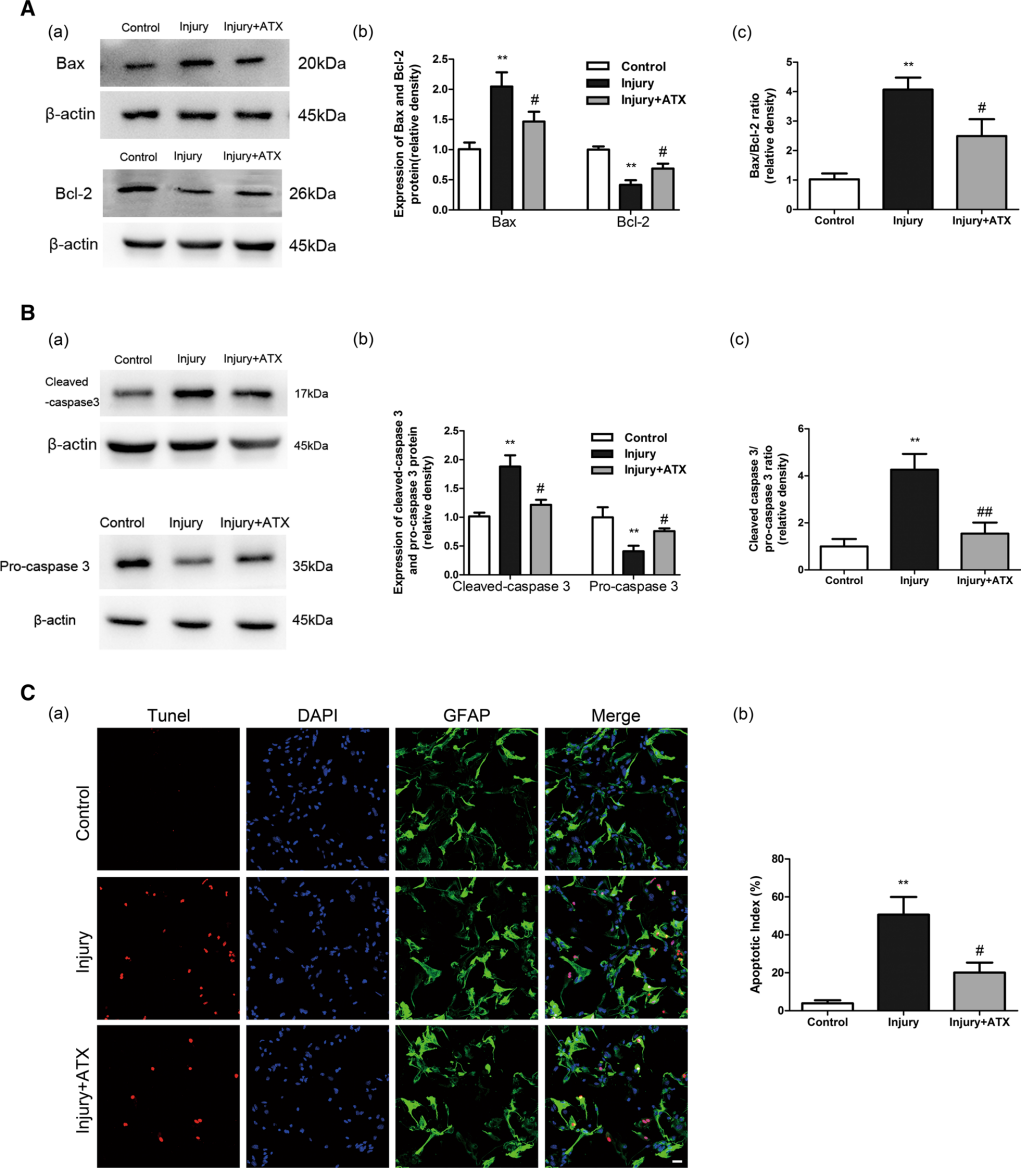
ATX inhibits the apoptotic cell death of astrocytes after injury. Injury caused a significant increase in the Bax/Bcl-2 ratio (**A**) and cleaved caspase-3/pro-caspase 3 ratio (**B**) in astrocytes, which was significantly attenuated by pretreatment with ATX. (**C**) Representative images of DAPI staining and TUNEL assays used to analyze apoptotic cells (magnification ×200). Quantification showed that pretreated with ATX markedly decreased the apoptotic index after injury. *Scale bar* 25 μm. *ATX* astaxanthin. Mean ± SEM (n = 3). **p < 0.01 versus the control group; ^#^ *p* < 0.05, ^##^ *p* < 0.01 versus the injury group

The protein levels of Bax, Bcl-2, cleaved caspase-3, and pro-caspase 3 were determined by Western blot analysis to establish if ATX is acted by regulating apoptotic proteins post-injury. Astrocytes were pretreated with ATX (50 μmol/L) and then injured and incubated for another 24 h. As demonstrated in Fig. 2A, Bax expression was significantly increased and Bcl-2 expression was markedly decreased after injury in comparison to the control group (*p* < 0.01), while pretreatment with ATX inhibited the upregulation of Bax and the downregulation of Bcl-2 (*p* < 0.05). In accordance with the aforementioned findings, cleaved caspase-3 protein level was strongly induced and pro-caspase 3 protein level was remarkably inhibited after injury compared to the control group (*p* < 0.01), indicating the involvement of caspase-3 in astrocytes undergoing traumatic cell death (Fig. 2B). However, administration of ATX for 2 h prior to injury significantly downregulated cleaved caspase-3 expression and upregulated pro-caspase 3 expression (*p* < 0.05).

Further, the trauma-induced apoptosis of astrocytes was additionally verified via the identification of TUNEL-positive staining in situ. As shown in Fig. 2C, the number of apoptotic cells was markedly increased in comparison to untreated cells, and the increased proportion of apoptotic cells was partially attenuated in the ATX-treatment group (50 μmol/L).

These outcomes show ATX successfully obstructs apoptotic cell death post-injury.

### Astaxanthin down-regulates the expression of IL-1β, IL-6 and TNF-α in astrocytes after injury

**Fig. 3:**
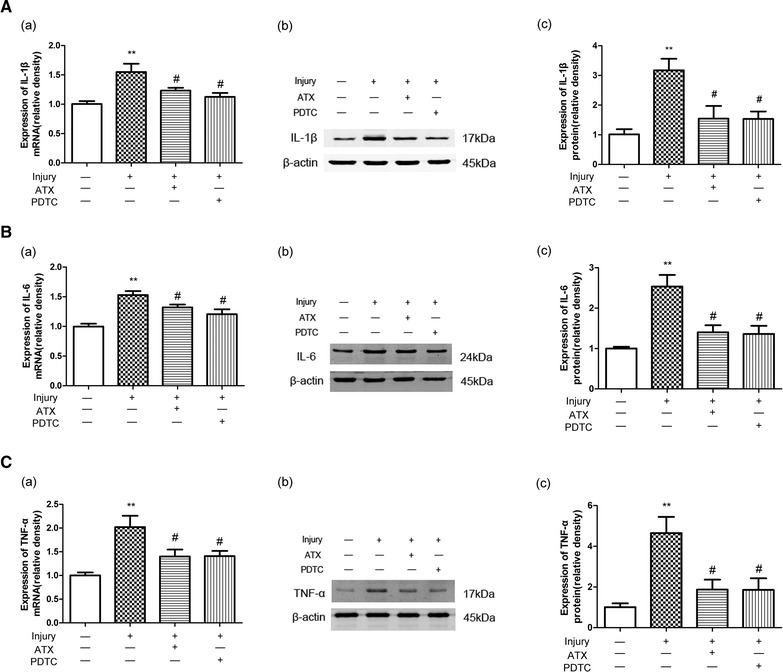
The effects of ATX on the mRNA and protein expressions of IL-1β (**A**), IL-6 (**B**) and TNF-α (**C**) in astrocytes after injury. Astrocytes were pretreated with ATX (50 μmol/L) or PDTC (10 μmol/L) for 2 h, injured, and incubated for 24 h. The mRNA expressions of IL-1β, IL-6, and TNF-α were elevated after injury compared with controls. However, the production of pro-inflammatory mediators was significantly reduced by ATX or PDTC. GAPDH was used as an internal control. A similar effect was observed for their protein expressions. *ATX* astaxanthin. Mean ± SEM (n = 3). **p < 0.01 versus the control group; ^#^ *p* < 0.05 versus the injury group

Pro-inflammatory cytokines, which include IL-1β, IL-6, and TNF-α, are believed to moderate neuroinflammation and cause cell death in different neurodegenerative diseases [24]. Thus, we studied the mRNA and protein expressions of pro-inflammatory factors by qPCR and Western blot analyses after injury. We pretreated astrocytes with ATX (50 μmol/L) for 2 h and then injured and incubated the cells for 24 h. As shown in Fig. 3, both mRNA expressions and protein levels of IL-1β, IL-6, and TNF-α were elevated after injury compared with controls (*p* < 0.01). However, the production of pro-inflammatory mediators was significantly reduced by ATX (*p* < 0.05). Furthermore, PDTC (10 μmol/L), an inhibitor of NF-κB, also significantly inhibited the expressions of IL-1β, IL-6 and TNF-α after injury (*p* < 0.05).

### Astaxanthin inhibits activation of the NF-кB pathway in astrocytes after injury

**Fig. 4:**
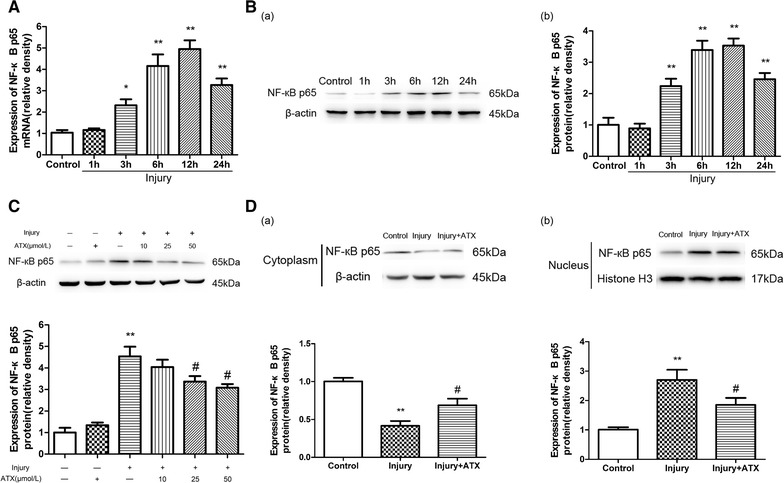
ATX inhibits activation of the NF-кB pathway in astrocytes after injury. Injury increased the mRNA (**A**) and protein expression (**B**) of NF-кB p65 in astrocytes in a time-dependent manner. **C** ATX (25 and 50 μmol/L) had a significant inhibitory effect on the expression of NF-кB p65 in astrocytes after injury. **D** NF-кB p65 expressions in the cytoplasm and nuclei were measured by Western blot. *ATX* astaxanthin. Mean ± SEM (n = 3). *p < 0.05, **p < 0.01 versus the control group; ^#^ *p* < 0.05 versus the injury group

Prior studies have revealed that the initiation of NF-кB controls the expression of inflammatory genes encoding pro-inflammatory cytokines (IL-1β, IL-6, and TNF-α) [25, 26]. Next, we investigate the expression of NF-кB mRNA and protein using qPCR and Western blot analyses at 1, 3, 6, 12, or 24 h after injury. As shown in Fig. 4A, B, both mRNA expression and protein levels of NF-кB were increased significantly after 3 h (*p* < 0.05 and <0.01, respectively) compared to control cells, with levels peaking at 12 h (*p* < 0.01) and lasting for 24 h (*p* < 0.01).

Then, Western blot analysis was performed to investigate the impacts of ATX on the upregulation of NF-кB proteins in astrocytes post-injury. Cells were pretreated with different concentrations of ATX for 2 h, injured, and then incubated for 24 h. ATX at 25 or 50 μmol/L showed a significant inhibitory effect on the raised expression of NF-κB (*p* < 0.05, Fig. 4C).

Nuclear and cytosolic extracts harvested from astrocytes after injury in the presence or absence of ATX were isolated, and Western blot analysis showed the nuclear translocation of NF-кB was elevated at 24 h after injury (*p* < 0.01). Pre-incubation with ATX (50 μmol/L) for 2 h prior to injury significantly decreased the level of NF-кB protein that translocated into the nucleus (*p* < 0.05, Fig. 4D).

### Astaxanthin down-regulates NKCC1 expression via the NF-кB pathway in astrocytes after injury

**Fig. 5:**
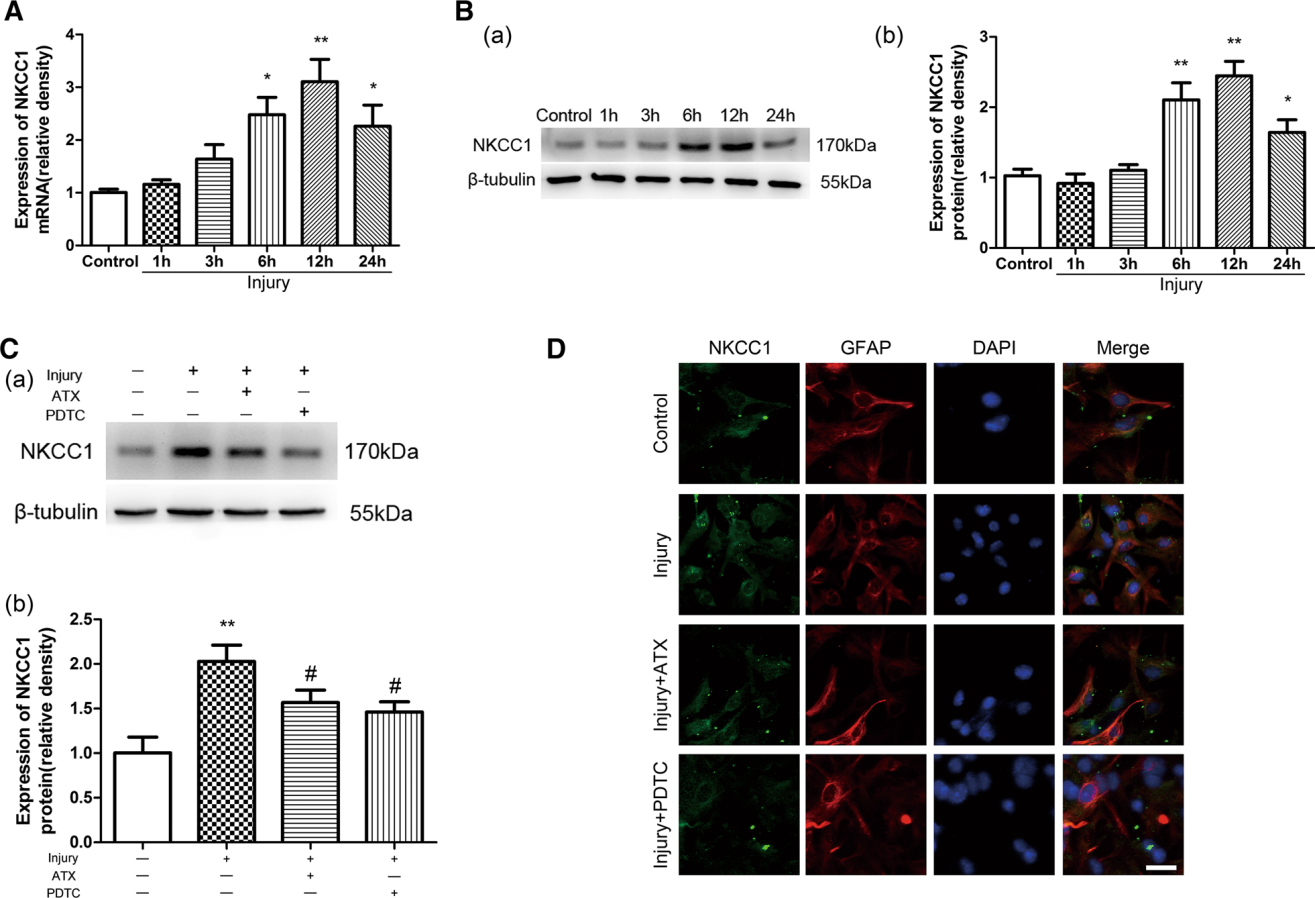
ATX or PDTC pretreatment inhibits NKCC1 expression in astrocytes after injury. NKCC1 mRNA level (**A**) and protein expression (**B**) were elevated in a time-dependent manner after injury. Pretreatment with ATX (50 μmol/L) or PDTC (10 μmol/L) for 2 h prior to injury reduced NKCC1 protein expression remarkably (**C**). Immunofluoresence was performed at 24 h after injury and the fluorescence intensity of NKCC1 in astrocytes was enhanced. Administration of ATX (50 μmol/L) or PDTC (10 μmol/L) for 2 h prior to injury decreased NKCC1 expression (**D**). *Scale bar* 25 μm. ATX, astaxanthin. Mean ± SEM (n = 3). *p < 0.05, **p < 0.01 versus the control group; ^#^ *p* < 0.05 versus the injury group

As shown in Fig. 5A, the NKCC1 mRNA level was elevated at 6 h after injury (*p* < 0.05), reaching a peak at 12 h (*p* < 0.01), and lasting for 24 h (*p* < 0.05). The protein expression of NKCC1 was similar after injury (Fig. 5B). However, pretreatment with ATX (50 μmol/L) or PDTC (10 μmol/L), a NF-кB inhibitor, significantly reduced the protein expression (Fig. 5C, *p* < 0.05).

A comparable effect was observed for NKCC1 expression by immunofluorescence staining (Fig. 5D). NKCC1 expression was significantly increased at 24 h after injury, while administration of ATX (50 μmol/L) or PDTC (10 μmol/L) decreased its expression compared to the injured group.

### Astaxanthin protects against injury-induced astrocyte apoptosis by inactivating the NF-κB/NKCC1 pathway

**Fig. 6:**
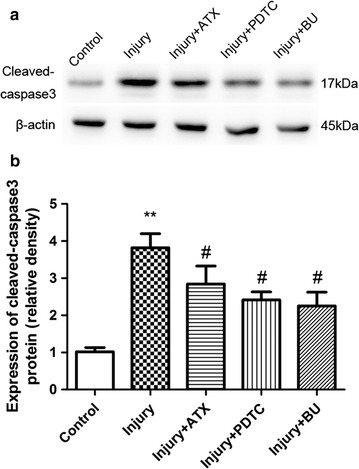
Neuroprotection by ATX-mediated inhibition of injury-induced cleaved caspase-3 expression is mediated by NF-κB and NKCC1 inactivation. **a** Injury significantly increased cleaved caspase-3 protein levels, while pretreatment with ATX (50 μmol/L) for 2 h significantly downregulated the elevated cleaved caspase-3 protein levels. Similarly, pretreatment with PDTC (10 μmol/L) or bumetanide (BU) (50 μmol/L) for 2 h prior to injury also markedly inhibited the increase in cleaved caspase-3 protein levels. **b** Densities of the corresponding bands were measured, and the ratios were calculated. Mean ± SEM (n = 3). **p < 0.01 versus the control group; ^#^ *p* < 0.05 versus the injury group

Last, we explored the possible roles of PDTC and bumetanide (inhibitors of NF-κB and NKCC1, respectively) in apoptosis of astrocytes after injury. The results showed that pretreatment with PDTC (10 μmol/L) and bumetanide (50 μmol/L) for 2 h significantly inhibited the increase in cleaved caspase-3 protein expression in astrocytes after injury (Fig. 6).

Altogether, these results suggest that ATX protects astrocytes from apoptotic cell death and these neuroprotective outcomes may be linked to the repression of NF-κB activation and later diminished activation of NKCC1.

## Discussion

The present study used an in vitro astrocyte stretch injury model to demonstrate that stretch injury directly decreased cell viability, increased apoptotic cell death and NKCC1 expression in cultured astrocytes. Furthermore, ATX, a naturally occurring carotenoid, inhibited these effects in a dose-dependent manner by decreasing the levels of pro-inflammatory cytokines activated by the NF-кB pathway after TBI. To the best of our knowledge, our results show for the first time, the relationship between ATX and NKCC1, a hallmark of cerebral edema, in an in vitro TBI model and the underlying regulatory mechanisms involved. Thus, ATX may be a novel therapeutic agent for the treatment of TBI.

Brain edema is one of the major consequences of TBI, and astrocyte swelling (cytotoxic edema) represents an important component of brain edema in the early phase [27]. Recent literature reported that in vitro trauma results in cell swelling and a higher susceptibility to cell death in cultured astrocytes [28, 29]. In the present study, the cytoactivity of astrocytes was measured after injury using CCK-8 assays. Cell viability was decreased in a time-dependent manner and reached a minimum at 24 h following injury. Nevertheless, pretreatment with various concentrations of ATX substantially improved this decrease in cell viability. Likewise, morphological changes that were representative of astrocyte damage were noted post-injury, while pretreatment with ATX somewhat reduced injury-induced cell damage. There exist two important pathways of apoptosis: the extrinsic pathway (receptor-mediated apoptotic pathway) and the intrinsic pathway (mitochondria-mediated apoptotic pathway) with possible cross talk. Numerous studies suggest that trauma-induced cell death transpires via the activation of apoptotic pathways [29, 30]. The cellular proteins in the innate pathway of apoptosis are part of the Bcl-2 and caspase families. It has been proposed that trauma causes cellular apoptosis by the buildup of Bax and the activation of caspase-3 [30]. Thus, the suppression of pro-apoptotic Bax expression or activated caspase-3 activity and the upregulation of anti-apoptotic Bcl-2 expression could be linked with neuroprotective effects against trauma. In agreement with these outcomes, we showed that trauma led to a rise in the Bax/Bcl-2 ratio and cleaved caspase-3/pro-caspase 3 ratio in cultured astrocytes. This effect, however, was substantially diminished by ATX, implying that it may protect astrocytes from traumatic injury via the regulation of apoptosis.

Inflammation is linked with astrocyte death and numerous pro-inflammatory mediators, including IL-1β, IL-6, and TNF-α, could be detrimental to astrocytes [31]. Further, TBI can prompt and inflate inflammatory reactions by their involvement in particular signaling pathways, including the NF-кB pathway [32]. Activation of the NF-кB pathway can cause a rise in the expression of many genes involved in inflammatory responses, such as cytotoxic cytokines (IL-1β, IL-6, and TNF-α), causing their raised expressions in tissues and direct cytotoxic effects [33]. Therefore, it is necessary to establish if stretch injury changes the expression of cytokine genes and if it is linked with the activation of NF-кB. We showed that mRNA and protein levels of IL-1β, IL-6, and TNF-α were increased in cultured astrocytes at 24 h after stretch injury. However, pretreatment with 50 μmol/L ATX reversed the trauma-induced increase of IL-1β, IL-6, and TNF-α mRNA and protein expressions. A comparable outcome was noted when we implemented PDTC, an inhibitor of NF-κB. This implies that ATX prompts its neuroprotective effects by suppressing pro-inflammatory cytokines, and these anti-inflammatory outcomes may halt cell death induced by trauma.

NF-кB consists of a family of transcription factors that positively regulate the expression of genes involved in inflammatory and other responses by binding to their promoters [34, 35]. Typically, the idle form of NF-кB stays in the cytoplasm as a heterodimer of p50 and p65 subunits. Its activation results in the dissociation and translocation of p65 and p50 subunits to the nucleus, where they up-regulate the transcription of pro-inflammatory genes [36]. We showed that NF-κB expression was significantly increased after stretch injury in a time-dependent manner and that increased levels of NF-кB were consistent with and relevant to the upregulation of IL-1β, IL-6, and TNF-α. Further, stretch injury prompted NF-кB translocation from the cytoplasm to the nucleus; however, ATX substantially prevented raised NF-κB expression and reduced injury-induced NF-κB translocation into the nucleus. A previous study showed that ATX reduced neuronal apoptosis in the cerebral cortex after subarachnoid hemorrhage by downregulating increased NF-κB activity and the expression of inflammatory cytokines [8]. We additionally examined if the prevention of NF-κB activation and translocation to the nucleus are involved in the mechanism of ATX antagonism of injury-induced apoptosis of astrocytes. The increased protein levels of cleaved caspase-3 were notably lowered by pretreatment with ATX and PDTC in astrocytes following stretch injury. Therefore, the activation of NF-кB could play a critical part in the trauma-induced apoptosis of astrocytes. Furthermore, ATX exhibited neuroprotective effects by inhibiting NF-κB activation and subsequently decreasing the upregulation of pro-inflammatory cytokines.

NKCC1, a membrane protein that allows the passage of ions and water through the cell membrane, is implicated in the formation of brain edema caused by various injurious insults in vivo, including TBI [14–16]. It was also involved in astrocyte swelling/brain edema in cultured astrocytes after fluid percussion injury [22]. The current study showed that NKCC1 mRNA and protein expression in primary astrocytes were significantly augmented in a time-dependent manner when subjected to stretch injury. However, the administration of 50 μmol/L ATX for 2 h before injury significantly ameliorated NKCC1 protein levels. This further supports the relationship between NKCC1 and astrocytes following in vitro trauma and suggests ATX might be a potent inhibitor of NKCC1. Furthermore, NF-кB activation was strongly implicated in the stimulation of various ion channels/exchangers in different conditions [37–40] and in the regulation of post-traumatic astrocyte swelling/brain edema [3]. Jayakumar et al. [27] reported a significant increase in NKCC activity after trauma to cultured astrocytes, and NF-кB inhibitor BAY-11 7082 blocked this activity, suggesting NF-кB-mediated cell swelling after trauma is, in part, a consequence of increased NKCC activity. Our data illustrate that NKCC1 expression was associated with increased NF-кB and that PDTC reduced NKCC1 expression. Therefore, ATX might reduce NKCC1 expression through inhibition of the NF-кB pathway. Kim et al. reported that NKCC was a good target for the induction of cell apoptosis by the activation of intracellular Ca^2+^ signaling and that the NKCC inhibitors, bumetanide and furosemide, markedly suppressed this effect [41]. We further examined whether the inhibition of NKCC1 activation was also involved in the antagonistic effect of ATX on the apoptosis of astrocytes after stretch injury. Pretreatment with bumetanide (50 μmol/L) significantly downregulated the elevated cleaved caspase-3 protein level in astrocytes, suggesting ATX reduced the trauma-induced apoptosis of astrocytes by inhibiting NKCC1 activation.

In previous study, we demonstrated that ATX might exert neuroprotection by ameliorating NKCC1-mediated cerebral edema after TBI in mice [11]. In this study, we elucidated that ATX prevented NKCC1 expression by limiting NF-κB-mediated pro-inflammatory mediators with an in vitro astrocyte stretch injury model. The discoveries recorded in this research paper, highlighting the anti-inflammatory role of ATX after trauma in cultured astrocytes, is consistent with its application in other diseases [8, 42–44]. Because ATX has other pharmacological properties, such as antioxidative and immunomodulatory properties, future studies should validate these properties in a TBI model. Further, NF-кB activation is regulated by a broad variety of upstream signals, including MAPKs [45–47], and thus, extensive studies should be performed to explore the explicit mechanisms.

## Conclusions

In summary, our findings indicate that ATX attenuates apoptosis after trauma in cultured astrocytes by inhibiting NKCC1 expression, and that it acts by reducing NF-κB-mediated pro-inflammatory factors. Therefore, ATX is an effective anti-inflammatory agent and might possess therapeutic prospects for TBI treatment.

## Abbreviations

ATX: astaxanthin
CCK-8: Cell Counting Kit-8
DAPI: 4′,6-diamidino-2-phenylindole
DMSO: dimethyl sulfoxide
GFAP: glial fibrillary acidic protein
IL: interleukin
NF-кB: nuclear factor kappa B
NKCC: Na^+^–K^+^–2Cl^−^ co-transporter
OD: optical density
PBS: phosphate buffered saline
PDTC: pyrrolidine dithiocarbamate
SEM: standard error of the mean
TBI: traumatic brain injury
TUNEL: TdT-dUTP nick-end labeling
WST-8: 2-(2-methoxy-4-nitrophenyl)-3-(4-nitrophenyl)-5-(2,4-disulfophenyl)-2*H*-tetrazolium and monosodium salt
